# Scbean: a python library for single-cell multi-omics data analysis

**DOI:** 10.1093/bioinformatics/btae053

**Published:** 2024-01-30

**Authors:** Haohui Zhang, Yuwei Wang, Bin Lian, Yiran Wang, Xingyi Li, Tao Wang, Xuequn Shang, Hui Yang, Ahmad Aziz, Jialu Hu

**Affiliations:** School of Computer Science, Northwestern Polytechnical University, 710129 Xi'an, Shaanxi, China; School of Computer Science, Northwestern Polytechnical University, 710129 Xi'an, Shaanxi, China; School of Computer Science, Northwestern Polytechnical University, 710129 Xi'an, Shaanxi, China; School of Computer Science, Northwestern Polytechnical University, 710129 Xi'an, Shaanxi, China; School of Computer Science, Northwestern Polytechnical University, 710129 Xi'an, Shaanxi, China; School of Computer Science, Northwestern Polytechnical University, 710129 Xi'an, Shaanxi, China; School of Computer Science, Northwestern Polytechnical University, 710129 Xi'an, Shaanxi, China; School of Life Science, Northwestern Polytechnical University, 710072 Xi'an, Shaanxi, China; Population Health Sciences, German Center for Neurodegenerative Diseases (DZNE), 53127 Bonn, Germany; Department of Neurology, Faculty of Medicine, University of Bonn, 53105 Bonn, Germany; School of Computer Science, Northwestern Polytechnical University, 710129 Xi'an, Shaanxi, China; Population Health Sciences, German Center for Neurodegenerative Diseases (DZNE), 53127 Bonn, Germany

## Abstract

**Summary:**

Single-cell multi-omics technologies provide a unique platform for characterizing cell states and reconstructing developmental process by simultaneously quantifying and integrating molecular signatures across various modalities, including genome, transcriptome, epigenome, and other omics layers. However, there is still an urgent unmet need for novel computational tools in this nascent field, which are critical for both effective and efficient interrogation of functionality across different omics modalities. Scbean represents a user-friendly Python library, designed to seamlessly incorporate a diverse array of models for the examination of single-cell data, encompassing both paired and unpaired multi-omics data. The library offers uniform and straightforward interfaces for tasks, such as dimensionality reduction, batch effect elimination, cell label transfer from well-annotated scRNA-seq data to scATAC-seq data, and the identification of spatially variable genes. Moreover, Scbean’s models are engineered to harness the computational power of GPU acceleration through Tensorflow, rendering them capable of effortlessly handling datasets comprising millions of cells.

**Availability and implementation:**

Scbean is released on the Python Package Index (PyPI) (https://pypi.org/project/scbean/) and GitHub (https://github.com/jhu99/scbean) under the MIT license. The documentation and example code can be found at https://scbean.readthedocs.io/en/latest/.

## 1 Introduction

With the rapid evolution of single-cell sequencing technologies over the past decade, single-cell omics data has emerged as a valuable resource for investigating numerous fundamental questions in biology. This includes the exploration of gene regulation (Van de Sande *et al.* 2020), the characterization of cellular diversity (Skelly *et al.* 2018), and the study of cell differentiation (Rizvi *et al.* 2017), among others (Hu *et al.* 2022b).

These technologies can be broadly categorized into two groups. The first group focuses on measuring the expression of a single modality at the single-cell level (Tang *et al.* 2009, Luo *et al.* 2018). The second, more advanced category enables the simultaneous measurement of two or more modalities within the same cell, exemplified by techniques, such as CITE-seq (Stoeckius *et al.* 2017). Owing to their remarkable high-throughput and high-resolution capabilities, numerous single-cell datasets have been generated, and substantial single-cell atlases have been constructed (Regev *et al.* 2017, Han *et al.* 2018). These resources have been pivotal in addressing fundamental inquiries in the field of life sciences, ranging from cell development to the comprehension of complex diseases.

However, the computational challenge of analyzing multi-omics data persists, primarily due to the presence of diverse statistical characteristics and batch effects that are inherent to various technologies and modalities (Zheng *et al.* 2021). The statistical features inherent in single-cell multi-omics data encompass high dimensionality, sparsity induced by a low capture rate of transcripts, and intrinsic noise. A substantial portion of non-biological variations in single-cell data can be attributed to batch effects, introduced through distinct sequencing technologies or diverse experimental conditions during library preparation and sequencing. However, effectively accounting for these non-biological sources of variations remains a formidable challenge. Addressing these challenges necessitates the application of sophisticated statistical models and machine-learning approaches to discern biological signals from noise. There is an immediate and pressing need for the development of an integrated tool designed to seamlessly handle the integration of single-cell multi-modal data. Such a tool should be capable of accommodating both unpaired and paired data, enabling a comprehensive exploration of cell heterogeneity, biological states, cell development, and spatial patterns within complex tissues.

Numerous methods have been proposed to analyze single-cell data, yielding promising outcomes. For unpaired data, several notable approaches have been developed, such as Seurat v3 (Stuart *et al.* 2019), LIGER (Welch *et al.* 2019), along with its enhanced versions like iNMF (Gao *et al.* 2021), VIPCCA (Hu *et al.* 2022a), DAVAE (Hu *et al.* 2021), scGLUE (Cao and Gao 2022), uniPort (Cao *et al.* 2022), and other deep learning-based methods (Zhang *et al.* 2022). These techniques have demonstrated their effectiveness in various applications and experimental setups. Simultaneously, several algorithms have been put forth for paired data, which encompass Seurat v4 (Hao *et al.* 2021), totalVI (Gayoso *et al.* 2021), and VIMCCA (Wang *et al.* 2023), along with various others. It is important to note that while these methods excel in many scenarios, they are often customized to specific modalities or application contexts. Additionally, these approaches have been implemented in various existing single-cell library toolkits, including Scanpy (Wolf *et al.* 2018), scvi-tools (Gayoso *et al.* 2022), and Seurat (Stuart *et al.* 2019).

To address these limitations, we have introduced a Python package named Scbean. This package offers a comprehensive set of computational tools capable of executing a wide range of integration tasks for both paired and unpaired single-cell data within a single, unified toolkit.

## 2 Implementation

Scbean is coded in the Python programming language, and a significant portion of its deep learning models is constructed upon the TensorFlow platform. Scbean is also equipped to harness GPU acceleration during the training phase, necessitating the installation of TensorFlow-gpu. In particular, we employed GPU acceleration to expedite the training process of our neural network models. This becomes particularly impactful when managing datasets comprising millions of cells.

**Figure 1.**
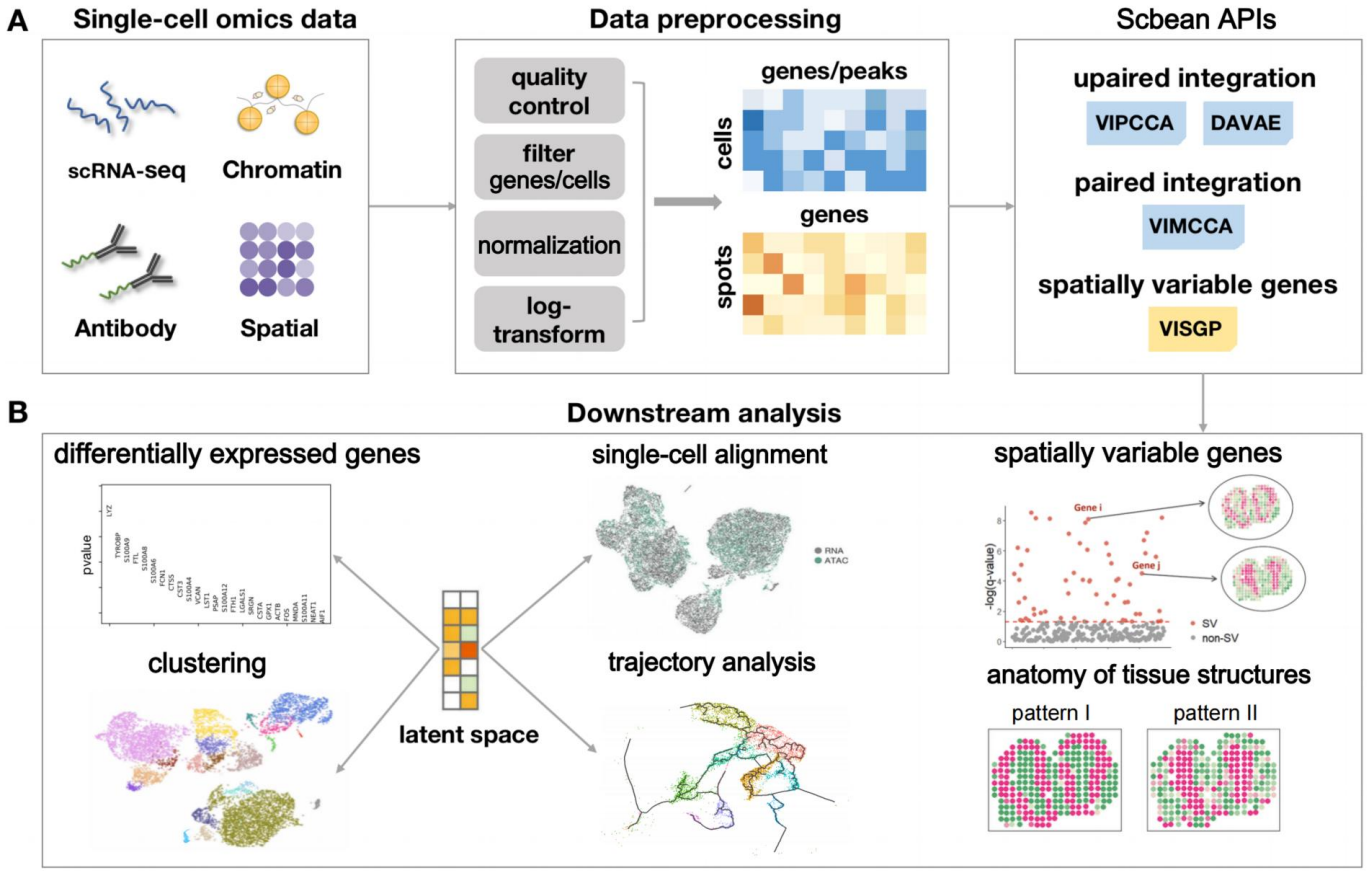
Overview of a multi-omics dataset pipeline employing Scbean. (A) Data collection, preprocessing, and loading data into APIs in Scbean. (B) The latent embedding obtained through the integration process can be leveraged for various subsequent analyses, including identification of marker genes, batch normalization, clustering, and trajectory analysis. Furthermore, results generated by VISGP can be instrumental not only in identifying spatially variable genes but also in elucidating the tissue’s structural anatomy.

The package offers users access to four distinct application programming interfaces (APIs), namely VIPCCA, DAVAE, VIMCCA, and VISGP (as shown in Fig 1). VISGP is optimized to handle moderately large-scale spatially resolved transcriptomic data, whereas the other three methods excel in managing large-scale single-cell omics datasets, often exceeding 1 million cells. All four APIs are crafted in a structured and consistent programming style, facilitating straightforward extensions.

Furthermore, Scbean is characterized by its user-friendly framework and robust compatibility with a wide range of other analysis tools. It seamlessly integrates with data preprocessed by Scanpy, and *vice versa*. The integrated data produced by Scbean remains accessible for a multitude of downstream analyses utilizing Scanpy, thanks to the common AnnData data type shared between Scbean and Scanpy.

Scbean’s statistical models are engineered to account for modality-specific variance sources, thereby augmenting the quality and precision of single-cell data integration. Additionally, single-cell data integrated by Scbean can be conveniently converted into R format via SeuratDisk, enabling downstream analyses utilizing the Seurat package.

## 3 Applications

**Figure 2.**
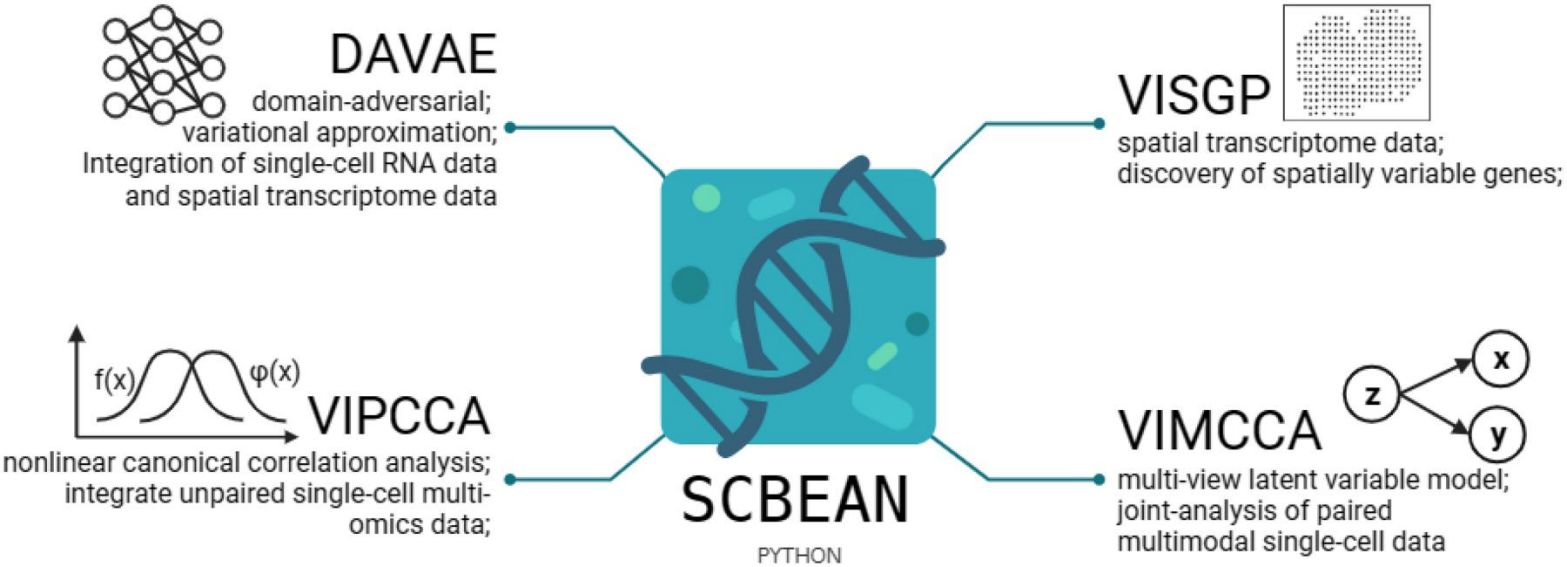
Four APIs of SCBEAN. DAVAE supports integration of scRNA-seq, scATAC-seq, and spatial transcriptomics based on domain-adversarial and variational approximation. VIPCCA supports integration of unpaired single-cell multi-omics data, differential gene expression analysis based on non-linear canonical correlation analysis. VIMCCA supports joint-analysis of paired multi-modal single-cell datasets based on a multi-view latent variable model. VISGP supports the discovery of spatially variable genes exhibiting distinct expression patterns in spatial transcriptomic data.

The Scbean package’s APIs serve as versatile tools for various facets of single-cell data analysis (as shown in Fig 2). VIPCCA is a model based on non-linear canonical correlation analysis, which first obtains an estimated dataset-specific embedding ${\hat{Z}}_{i}^{m}$ (for the $i^{th}$cell and the $m^{th}$ batch) in the low dimensional space through variational approximation and then pairs it with the dataset-specific annotation $b^{m}$ to recover the dataset-specific gene expression. VIPCCA excels in the effective reduction of batch effects within scRNA-seq datasets originating from different technologies. Besides, it possesses the capability to align scATAC-seq cells to a scRNA-seq dataset, which serves as a reference cell map. Another highly versatile API, DAVAE, is a model based on a combination of a domain-adversarial neural network and variational approximation. It facilitates integration of multiple omics data from different modalities, encompassing transposase accessible chromatin data, single nuclei transcriptomic data, and spatial transcriptomic data. VIMCCA is a model based on variational-assisted multi-view canonical correlation analysis, which is specially tailored for the integration of paired single-cell multi-modality data, exemplified by CITE-seq (RNA and protein) and 10X Genomics Multiome (RNA and ATAC). In addition to these integration tools, Scbean provides VISGP, designed based on a variational Gaussian process, which is able to adapt its shape to match complex posterior distributions. It can be used for the discovery of spatially variable genes exhibiting distinct expression patterns in spatial transcriptomic data. To expedite users in launching applications, Scbean’s documentation and tutorials offer detailed information, presenting example code alongside sample data, guiding users step-by-step. Moreover, the documentation covers installation instructions, preprocessing workflows, parameter settings, visualization techniques, and downstream analysis procedures in depth.

## 4 Results and discussion

To test the capabilities of our integrative package scbean in analyzing single-cell omics data, we test each of the four APIs on one real world dataset. First, we run VIPCCA for aligning single-cell omics data acquired from disparate measurement batches: a scRNA-seq dataset of 19 089 genes measured on 5140 cells and a scATAC-seq dataset of 89 796 peak measurements on 5234 nuclei (see Supplementary Text for more details). After the preprocessing of the raw data, VIPCCA calculates the embedding in the lower-dimensional space, and visualizes the results via UMAP. In the UMAP, each dot represents a cell/nucleus colored by datasets. VIPCCA successfully aligned these two disparate datasets without any complex specific algorithmic procedures. The UMAP visualization shows that VIPCCA mixes the two data types well (as shown in Supplementary Fig. S1). Overall, the above example demonstrates the ability of VIPCCA integrating scATAC-seq data with scRNA-seq data. In the second case study, we employed DAVAE to integrate a scRNA-seq smart-seq dataset and two spatial transcriptomic datasets derived from two slices of sagittal mouse brain tissues—the anterior and posterior portions—both profiled using the 10X/Visium technology. The two spatial datasets consist of a total of 32 285 genes measured across 2696 spots in the anterior slice and 3353 spots in the posterior slice, and the scRNA-seq data measured 36 577 genes on 22 272 cells. As shown in Supplementary Fig. S2, we successfully identified 26 clusters, in which we can clearly visualize the stratification of the cortical layer in both of the two tissues in spatial coordinates. In the third case study, we ran VIMCCA on a paired RNA+ATAC single-cell dataset obtained from Peripheral Blood Mononuclear Cells from a healthy donor (see Supplementary Text for more details). As shown in Supplementary Figs S3 and S4, all cell types can be clearly recognized in the UMAP visualization after the integration of the two modalities. In the last case, VIGSP was performed on a spatial transcriptomic dataset of human breast cancer (Layer 1), which contains 14 881 genes measured on 254 spots. In the preprocessing, we filtered out genes with <10 reads, and spots with 10 total read counts. From the results in Supplementary Fig. S5, we can see that these spatially specific genes identified by VISGP have distinct spatial expression patterns.

## 5 Conclusion

Scbean is a scalable toolkit that can perform many data analysis tasks, including dimensionality reduction, removing batch effects, and transferring well-annotated cell type labels from scRNA-seq to scATAC-seq and spatially variable genes. It integrates a range of models, including VIPCCA, DAVAE, VIMCCA, which is efficient and scalable to over millions of cells. Additionally, both paired and unpaired single-cell multi-modal datasets are considered. It can be seamlessly integrated into existing analysis pipelines, allowing users to perform downstream analysis using scanpy or convert the data to Rdata format for further analysis. Additionally, Scbean provides the VISGP method for identifying genes with spatial patterns that can potentially benefit the research community in assessing 3D tissue’s structure anatomy. To facilitate usage, detailed Jupyter notebook tutorials are provided, covering installation, parameter settings, model training, and result visualization. Scbean will also provide more fundamental analyses for multi-modal data and spatially resolved transcriptomic data in the future. Specifically, Scbean could potentially be improved in several directions. First, our focus will be on developing more robust tools for the analysis of multi-omics data, such as construction of gene regulatory networks. This endeavor aims to streamline the process of addressing fundamental biological questions with greater efficiency and precision. Secondly, Scbean will furnish foundational data structures and commonly utilized APIs. This provision aims to ensure accessibility for researchers with limited programming experience, facilitating user-friendly utilization of the tool. In summary, Scbean is an integrated tool that offers powerful methods and a user-friendly interface for single-cell data analysis and integration. It supports GPU acceleration, is compatible with existing tools, and provides comprehensive tutorials to assist users in exploring the complexity of single-cell data and unraveling cellular heterogeneity. We will continue to update and improve the framework and expect that scbean will find a wide range of applications in the field of integrated single-cell multi-omics data analysis.

## Supplementary Material

btae053_Supplementary_Data

## Data Availability

All datasets used in the case studies of DAVAE, VIPCCA, and VIMCCA can be accessed at the 10x Genomics Datasets (https://www.10xgenomics.com), while the data used for testing VISGP were downloaded from the Spatial Transcriptomics Research at https://www.spatialresearch.org. See Supplementary Text for more details.
