# Supplementary material for: Scbean: a python library for single-cell multi-omics data analysis
Source: Bioinformatics. 2024 Jan 30;40(2):btae053. doi: 10.1093/bioinformatics/btae053 (PMC10868338; doi:10.1093/bioinformatics/btae053)
Supplement: btae053_Supplementary_Data [file btae053_supplementary_data.pdf]

Scbean: a python library for single-cell multi-omics data analysis

Haohui Zhang[1], Yuwei Wang[1], Bin Lian[1], Yiran Wang[1], Xingyi Li[1], Tao Wang[1], Xuequn Shang[1], Hui Yang[2], Ahmad Aziz[3,4,*], and Jialu Hu[1,3,*]

[1]*School of Computer Science, Northwestern Polytechnical University, 1 Dong Xiang Rd., 710129, Shaanxi, China. Tel: 029-88431519.*
[2]*School of Life Science, Northwestern Polytechnical University, 127 West Youyi Rd., 710072, Shaanxi, China. Tel: 86-29-88460332.*
[3]*Population Health Sciences, German Center for Neurodegenerative Diseases (DZNE), Venusberg-Campus 1, Building 9, 53127, Bonn, Germany. Tel: 49-228-43302-954.*
[4]*Department of Neurology, Faculty of Medicine, University of Bonn, Venusberg-Campus 1, 53105, Bonn, Germany. Tel: 49-228-43302-954.*

1 Supplementary Text

1.1 Detailed information for applications on four real datasets

To test the capabilities of our integrative package scbean, we here demonstrate one case study for each API. First, we perform VIPCCA [1] for aligning single-cell omics data obtained from disparate measurement batches, which includes both scRNA-seq with scRNA-seq, as well as scRNA-seq with scATAC-seq. To exemplify the application of VIPCCA, we have chosen scRNA-seq and scATAC-seq data from peripheral blood mononuclear cells (PBMCs) derived from two healthy human donors for experimentation. As VIPCCA requires equivalent numbers of features for input data, the first course of action is data preprocessing to select highly variable genes. It include three major steps: 1) Utilizing scanpy.pp.filter_genes and scanpy.pp.filter_cells

*Correspondence to J.H.: jhu@nwpu.edu.cn

to eradicate low-quality genes (peaks) and cells; 2) Implementing CPM and logarithmic normalization with scanpy.pp.normalize and scanpy.pp.log1p; 3) Selecting the top 2000 highly specific genes and peaks from RNA and ATAC data using scanpy.pp.highly_variable function. After the preprocessing, the VIPCCA function in the Scbean is used for data alignment. The four hyperparameters epochs, lambda_regulizer, batch_input_size, batch_input_size2 are user-defined parameters. Given a dataset with 10K cells, we suggest to pick up a value larger than 600 for epochs, a value in the range of [2,10] for lambda_regulizer, a value at least less than the number of input features for batch_input_size, a value in the range of [8,16] for batch_input_size2. The aligment result is shown in Fig. S1.

In the second case, we use a 10X genomics dataset as an example to explore DAVAE's [2] various functionalities. We test it on mixed-cell-line datasets from 10x genomics with non-overlapping populations from three batches, two of which contain 293t (2885 cells) and jurkat (3258 cells) cells respectively, and the third batch contains a 1:1 mixture of 293t and jurkat cells (3388 cells). Use the read_sc_data function in scbean.tools.utils to load the data sets and load each data set into an AnnData object. Then call davae.fit_integration, which contains four user-defined hyperparameters, batch_num, domain_lambda, epochs, hidden_layers. In practice, we chose the hidden layer parameters to be [64, 32, 6]. Then, an AnnData object is returned into an anndata object and the meta.data of each cell has been saved in adata.obs and the embedding representation from vipcca of each cell have been saved in adata.obsm('X_davae'). In the downstream analysis, we use UMAP to reduce the embedding feature output by davae in two dimensions and visualize its results (see in Fig. S2). In addition, DAVAE has the ability to integrate spatial transcriptome data and integrate spatial transcriptome data with scRNA-seq data. More details can be found in the tutorials we provide. The documentation and example code can be found at https://scbean.readthedocs.io/en/latest/.

In the third case, VIMCCA [3] is leveraged to integrate scRNA-seq and scATAC-seq data simultaneously obtained from the same cell. In this experiment, the raw data, with the CSV format, is initially read via panda's read_csv function. The data is then converted into the AnnData format, the recommended input format for scbean, using the AnnData data structure of the scanpy library. Subsequently, users have options to preprocess the count data with scanpy, although this step is not mandatory. Alternatively, VIMCCA also supports the direct input of raw count data. Preprocessing can involve utilizing scanpy.pp.filter_genes and scanpy.pp.filter_cells to eliminate low-quality cells and genes, undertaking CPM and logarithmic normalization with scanpy.pp.normalize and scanpy.pp.log1p, and finally rendering the data

to standard distribution of zero mean and unit variance using scanpy.pp.scale. Upon completion of these processes, scbean.model.VIMCCA.fit_integration can be employed for data integration. In practice, we chose the hidden layer parameters to be [128, 64, 32, 16, 10]. Detailed information for code examples and workflow are depicted in Fig. S3. The UMAP visualization of the integration is shown in Fig. S4.

In the last case, we perform VISGP to spatial transcriptome data of human breast cancer (layer 1), which contains 14,881 genes measured on 254 spots. First of all, the data is read through the pd.read_csv function and goes through a series of pre-processing methods, including separating the x and y coordinates of spot and filtering these genes that are almost not observed. Then, we construct a matrix of gene*spots, and integrate the data into an anndata to facilitate data processing and data management. After that, the visgp.VISGP function is used to construct the object from the above data and the run function is called to perform the inference algorithm. Finally, the space-specific gene result will be calculated, which is in the form of a DataFrame with p-value and adjusted p-value (q-value) for each gene. We called these genes with q-value less than 0.05 as spatially variable genes. With these obtained spatially specific genes, some other packages such as xx can be used in the downstream analysis and visualize gene expression patterns. The analysis process usually includes the transformation of variance stabilization and linear standardization. The visualization of spatial patterns in this case can be seen in Fig. S5

1.2 Data availability

In our example cases , we use DAVAE to integrate an scRNA-seq data and a spatial gene expression data downloaded from the 10xGenomics Datasets at `https://www.10xgenomics.com/cn/resources/datasets?query=&page=1&configure%5BhitsPerPage%5D=50&configure%5BmaxValuesPerFacet%5D=1000`. The datasets utilized to demonstrate the alignment capabilities of VIPCCA consist of an scRNA-seq data of 5k-human-pbmcs-3-v3-1-chromium-controller-3-1-standard and an scATAC-seq data of 5-k-peripheral-blood-mononuclear-cells-pbm-cs-from-a-healthy-donor-next-gem-v-1-1-1-1-standard-1-1-0 in the 10x Genomics at `https://www.10xgenomics.com/resources/datasets/5k-\human-pbmcs-3-v3-1-chromium-controller-3-1-standard` and `https://www.10xgenomics.com/resources/datasets/5-k-peripheral-blood-mononuclear-cells-pb` seq profiles from PBMCs of two healthy human donors. We use VIMCCA to integrate data of Chromium Single Cell Multiome ATAC + Gene Expression assay, which was downloaded from `https://www.10xgenomics.com/support/single-cell-multiome-atac-plus-gene-expression` at the 10x

Genomics. The data used for testing VISGP was downloaded from `https://www.spatialresearch.org/resources-published-datasets/doi-10-1126science-aaf2403/` at the Spatial Transcriptomics Research.

2 Supplementary Figures

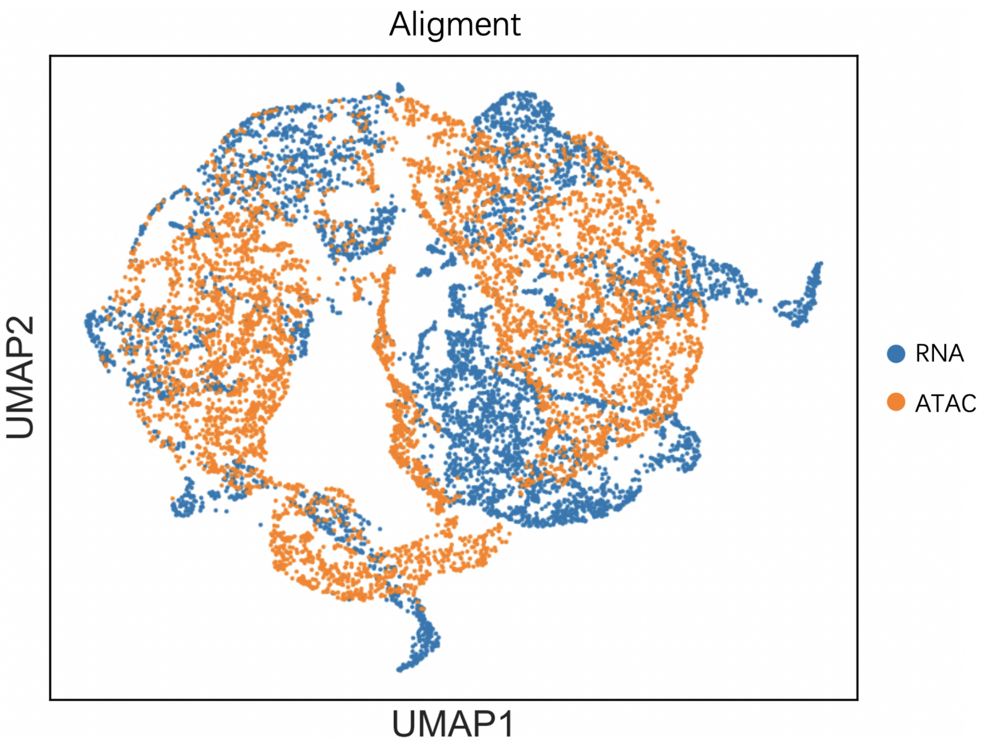

Figure S1: **UMAP visualization obtained by VIPCCA.**

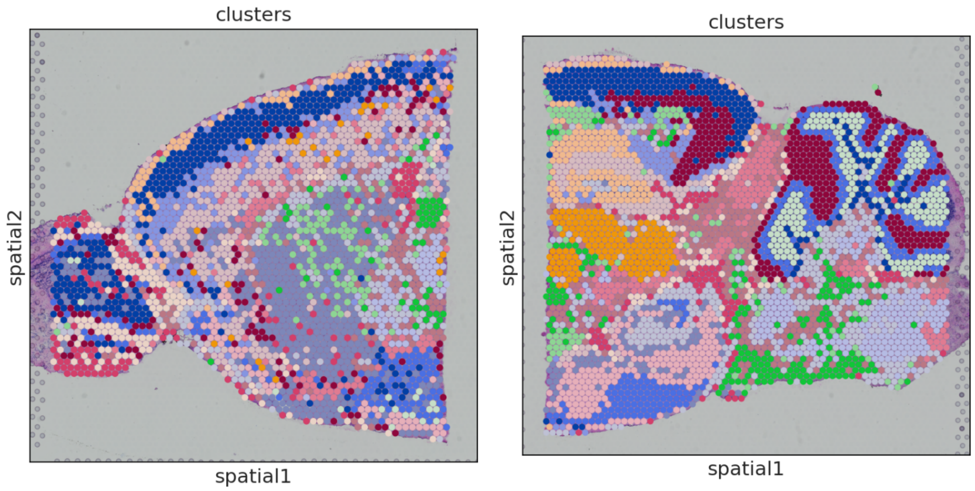

Figure S2: **Visualize the results of DAVAE in spatial coordinates.**

```python
import pandas as pd
import scanpy as sc
from scbean.model import vimcca

# Read original
rna_pd = pd.read_csv("/Users/wangyuwei/dataset/vimcca/rna.csv", index_col=0)
atac_pd  = pd.read_csv("/Users/wangyuwei/dataset/vimcca/atac.csv", index_col=0)

# Obtain AnnData
adata_rna = sc.AnnData(rna_pd.values)
adata_atac = sc.AnnData(atac_pd.values)

# Preprocessing data (optional)
## Filter genes and cells
sc.pp.filter_genes(adata_rna, min_cells=10)
sc.pp.filter_genes(adata_atac, min_cells=10)
sc.pp.filter_cells(adata_rna, min_genes=100)
sc.pp.filter_cells(adata_atac, min_genes=100)
## Normalization
sc.pp.normalize_total(adata_rna)
sc.pp.normalize_total(adata_atac)
sc.pp.log1p(adata_rna)
sc.pp.log1p(adata_atac)

# Integrate via VIMCCA
adata_rna.obsm['X_vimcca'] = vimcca.fit_integration(adata_rna, adata_atac,
                                    hidden_layers=[128, 64, 32, 16, 10],
                                    epochs=165,
                                    sparse_x=False,
                                    sparse_y=False)
# Visualization
sc.pp.neighbors(adata_rna, use_rep="X_vimcca")
sc.tl.umap(adata_rna)
sc.pl.umap(adata_rna, color="celltype")
```

Figure S3: **Code details for integrating scRNA-seq and scATAC-seq obtained from the same cell using VIMCCA.**

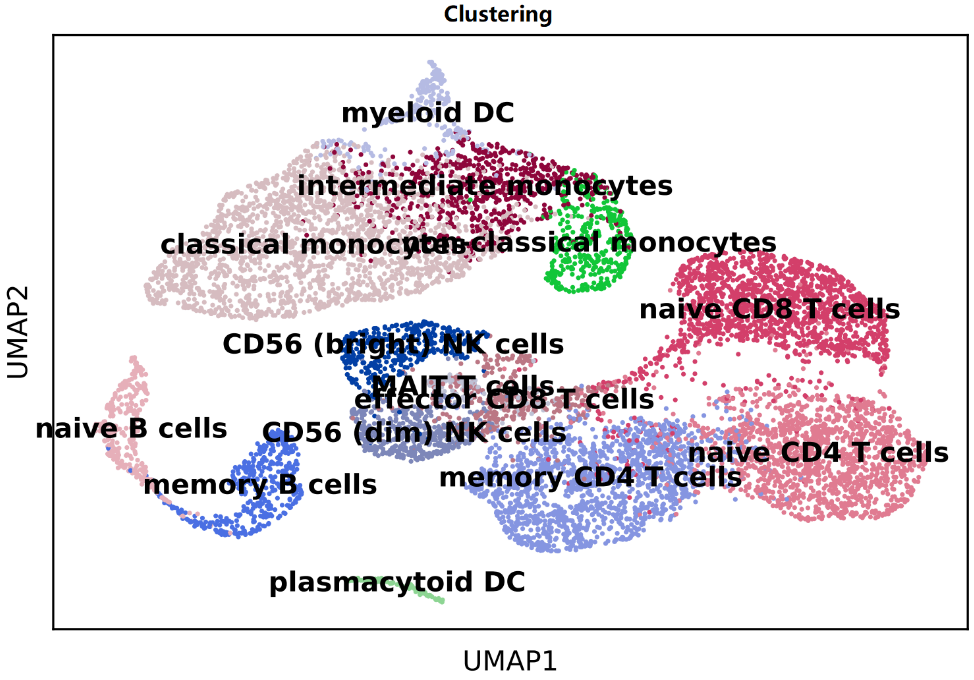

Figure S4: **UMAP visualization obtained by VIMCCA.**

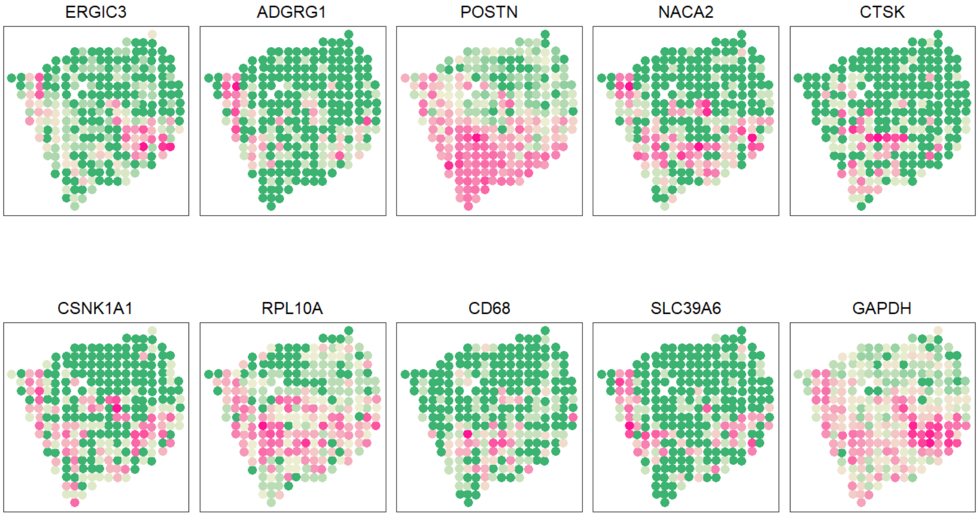

Figure S5: **Gene expression pattern obtained by VISGP.**

References

[1] Hu J, Chen M, Zhou X. Effective and scalable single-cell data alignment with non-linear canonical correlation analysis. Nucleic acids research. 2022;50(4):e21-1.

[2] Hu J, Zhong Y, Shang X. A versatile and scalable single-cell data integration algorithm based on domain-adversarial and variational approximation. Briefings in Bioinformatics. 2021 09;23(1). Bbab400. Available from: https://doi.org/10.1093/bib/bbab400.

[3] Wang Y, Lian B, Zhang H, Zhong Y, He J, Wu F, et al. A multi-view latent variable model reveals cellular heterogeneity in complex tissues for paired multimodal single-cell data. Bioinformatics. 2023;39(1):btad005.
